# Redefinition of dementia care in Italy in the era of amyloid-lowering agents for the treatment of Alzheimer’s disease: an expert opinion and practical guideline

**DOI:** 10.1007/s00415-023-11642-0

**Published:** 2023-03-09

**Authors:** Massimo Filippi, Giordano Cecchetti, Annachiara Cagnin, Camillo Marra, Flavio Nobili, Lucilla Parnetti, Federica Agosta

**Affiliations:** 1 Neurology Unit, IRCCS San Raffaele Scientific Institute, Via Olgettina, 60, 20132 Milan, Italy; 2 Neuroimaging Research Unit, Division of Neuroscience, IRCCS San Raffaele Scientific Institute, Via Olgettina, 60, 20132 Milan, Italy; 3 Vita-Salute San Raffaele University, Via Olgettina, 60, 20132 Milan, Italy; 4 Neurology Unit, Department of Neuroscience, University of Padova, Padua, Italy; 5 Padova Neuroscience Center (PNC), University of Padova, Padua, Italy; 6 Memory Clinic, IRCCS Policlinico Gemelli, Rome, Italy; 7 Department of Neuroscience, Università Cattolica del Sacro Cuore, Rome, Italy; 8 Neurology Unit, IRCCS Ospedale Policlinico San Martino, Genoa, Italy; 9 Department of Neuroscience (DINOGMI), University of Genova, Genoa, Italy; 10 Centre for Memory Disturbances, Section of Neurology, Lab of Clinical Neurochemistry, Department of Medicine and Surgery, University of Perugia, Perugia, Italy

**Keywords:** Expert opinion, Alzheimer, Italy, Disease-modifying therapies, Monoclonal antibodies

## Abstract

No disease-modifying therapies are currently available for Alzheimer’s disease (AD) in Europe. Current evidence from clinical trials testing anti-beta amyloid (Aβ) monoclonal antibodies (mAbs) in patients with early AD, though, suggests a likely marketing authorization in the next years. Since the implementation of disease-modifying therapies for AD in the clinical practice will evidently require a huge change of dementia care in all countries, a group of prominent AD clinical experts in Italy met to discuss patients’ selection and management strategies. The current diagnostic–therapeutic standard of care in Italy was taken as the starting point. The prescription of new therapies cannot ignore the definition of a biological diagnosis through the assessment of both amyloid- and tau-related biomarkers. The high risk/benefit ratio of anti-Aβ immunotherapies, moreover, needs a highly specialized diagnostic work-up and a thorough exclusion criteria assessment, which should be provided by a neurology specialist. The Expert Panel also suggests a reorganization of the Centers for dementia and cognitive decline in Italy into 3 levels of increasing complexity: community center, first- and second-level center. Tasks and requirements for each level were defined. Finally, specific characteristics of a center deputed to prescribe anti-Aβ mAbs were discussed.

## Introduction

After more than a century since the first description of clinical and neuropathological correlates of Alzheimer’s disease (AD), and after more than a decade of research, encouraging results of clinical trials testing anti-β amyloid (Aβ) monoclonal antibodies (mAbs) in patients with AD have been achieved. Currently, three anti-Aβ mAbs have ongoing or recently completed phase-3 trials in early AD, and they have been approved or are under examination for accelerated approval in the United States (US). An approval of one or more of them also in Europe in the forthcoming years is expected. [1–3]

In June 2021, based on the evidence of efficacious removal of cerebral amyloid plaques in EMERGE and ENGAGE studies, and despite the lack of definitively proven clinical benefit, the U.S. Food and Drug Administration (FDA) granted accelerated approval to Aduhelm^®^ (aducanumab, a human IgG1 anti-Aβ mAb, ascending intravenous doses to 10 mg/kg every 4 weeks–Biogen Inc.) for the treatment of mild cognitive impairment (MCI) due to AD and mild AD dementia [4]. Data from post-marketing studies will be crucial to determine whether continued approval will be warranted. On April 22, 2022, in light of a very unlikely marketing authorization, the company withdrew the application from the European Medicines Agency (EMA), and a phase-3b/4 confirmatory study (ENVISION trial) has been meanwhile implemented to possibly establish, by the end of 2026, a definite link between the biological effect (i.e., amyloid removal) and clinical improvement [5]. Encouraging results from phase-3 confirmatory Clarity AD clinical trial testing lecanemab (a humanized IgG1 anti-Aβ protofibril mAb, ascending intravenous doses to 10 mg/Kg biweekly–Eisai/Biogen Inc.) and from phase-2 TRAILBLAZER 1 study testing donanemab (a humanized IgG1 anti-Aβ mAb, ascending intravenous doses from 700 to 1400 mg every 4 weeks–Eli Lilly and Company), confirming a significant clinical effect, have been recently presented [6–8]. Indeed, on July 5, 2022, and August 4, 2022, the FDA formally accepted the company’s Biologics Licence Application (BLA) for lecanemab and donanemab, respectively, granting them priority reviews. On January 6, 2023, the agency approved lecanemab (Leqembi™) for the treatment of MCI due to AD and mild AD dementia [9], and on January 10, 2023, the company submitted marketing authorization application to the EMA [10]. A final decision of the FDA on donanemab is instead expected by early February 2023 [3]. On November 14, 2022, after having been granted breakthrough therapy designation by the FDA for the treatment of AD in October 2021, phase-3 clinical trials testing gantenerumab (a fully humanized IgG1 anti-Aβ mAb, ascending subcutaneous doses to 510 mg every 2 weeks–Hoffman-La Roche) were unfortunately discontinued based on the lack of evidence for clinical and biological efficacy in GRADUATE 1 and 2 studies [4, 11].

In light of this evidence, prominent clinical experts in Italy with complementary experience in AD clinical care, research, clinical trials, and diagnosis (including CSF, neuroradiology and nuclear medicine biomarkers) met to discuss major aspects regarding patient selection and to propose possible management strategies, should an anti-Aβ mAb obtain marketing authorization in our country. The introduction of an effective disease-modifying treatment for AD, indeed, will require enormous change in the delivery of dementia care [12]. The indications here included are based on the Italian scenario, but they could also be implemented in other European countries with the necessary adjustments related to country-specific peculiarities.

## The transition toward a biomarker-based biological diagnosis of AD

Aiming at the early diagnosis of AD to increase the effectiveness of therapeutic interventions, MCI was proposed as an intermediate stage of a continuum that ranges from normal cognitive functioning to AD dementia [13]. MCI is a syndrome characterized by a decline in cognitive performance greater than expected for an individual’s age and education level, but that does not interfere with daily life activities [13]. More recently, since the dementia syndrome does not necessarily assure a specific underlying neuropathology, on the verge of a possible approval of AD-specific disease-modifying therapies, the need for a biological rather than a syndromic definition of AD became evident. In 2018, the National Institute on Aging–Alzheimer’s Association (NIA-AA) responded to this need, proposing a set of research criteria for the biological diagnosis of AD that, regardless of clinical symptoms, requires evidence of abnormalities in both beta amyloid (Aβ) biomarkers (“A”: CSF Aβ, amyloid-PET) and phosphorylated tau (pTau) biomarkers (“T”: CSF pTau, tau-PET) [14]. The demonstration of “A” alone, indeed, defines the stage of cerebral amyloidosis without neurodegeneration and therefore a pre-AD stage; moreover, cerebral amyloidosis is not specific to AD as it is seen in other neurodegenerative diseases with amyloid copathology [15]. This framework allows, in case of both “A” and “T” abnormalities, the application of the label “AD” in any person within a continuum from normal cognitive functioning (defined “preclinical AD”), to MCI (“prodromal AD”) and finally dementia (“AD with dementia”). Moreover, a biological diagnosis is crucial in case of atypical phenotypes (posterior cortical atrophy, logopenic variant of primary progressive aphasia, behavioral/dysexecutive variant, corticobasal syndrome) to assure the demonstration of an AD underlying pathology [15]. “N” biomarker category (structural MRI, FDG-PET, and CSF total tau) was also proposed to define the presence of neuronal injury and to provide powerful tools to assess the risk of progression from preclinical to prodromal AD, and from prodromal AD to dementia [14]. More recently, different plasma-based biomarkers reflecting the whole spectrum of the A/T/N system (Aβ/pTau/NfL and GFAP) are under investigation for a possible future implementation in the clinical practice, potentially revolutionizing the way AD will be diagnosed and treated in the next future [16–20]. While there are still challenges and limitations to overcome, indeed, ongoing research and development in this area are likely to lead to significant advances in the coming years. As research continues, plasma biomarkers may become more reliable and accurate at detecting AD in its early stages (even before symptoms appear) and at tracking disease progression over time.

## Epidemiologic considerations

According to data presented in the ‘Dementia in Europe Yearbook 2019’, the number of people living with dementia in Italy was 1.3 million in 2018 and is expected to raise to 1.4–1.5 million by 2025, 50–70% of which facing AD (i.e., 0.7–1.1 millions) [21, 22]. With regard to MCI, many authors reported a prevalence of 15–20% in the general population, with a rate of conversion to dementia of 10–15% per year [23]. In a 2008 Italian study, the prevalence of MCI in a population over the age of 65 was 7.7% (51% of which of the amnestic type), meaning 1,081,958 people in Italy suffering from MCI (551,799 with the amnestic type) [24]. Taking a recent global estimation of the prevalence of amyloid-positive population as a good starting point for deriving relevant AD prevalence estimates [25], the number of individuals with prodromal AD in our country might even exceed the commonly cited estimates of AD burden, ranging from 663,530 to 1,478,953, whereas the prevalence of preclinical AD could range from the 12% of individuals of 50–54 years of age, to the 24% of people of 75–79 years of age.

By systematically applying the inclusion/exclusion criteria adopted in EMERGE and ENGAGE trials to patients referred to two high-specialized memory clinics in Italy, estimates of the proportion of eligible patients within the target MCI population ranged from the 0.7% in a geriatric clinic [26], to the 32.6% in a neurologic clinic (meaning ~ 45,000 patients nationally) [27]. Considering that the prescribing criteria in the real-world setting might be likely broader than those used in clinical trials [4], the number of eligible patients may be even greater. Notwithstanding contraindications or other restrictions to treatment eligibility, indeed, in a recent French review, Villain et al. estimated that more than 66,000 patients in France could be eligible for an anti-Aβ immunotherapy, and that this value might be more likely 5- to 10-fold higher [2]. Based on these considerations, it is reasonable to assume that the number of prodromal and mild AD patients eligible for amyloid-lowering therapies in Italy will not be dissimilar; it could even double in the future, in the hypothetical case that access to therapies is granted also to those preclinical AD subjects that are at higher risk to evolve to prodromal AD on the basis of prognostic biomarkers [28, 29].

## Patient selection

The prescription of anti-Aβ mAbs cannot be separated from the confirmation of AD pathology, requiring, as previously stated, the positivity of biomarkers of both amyloidopathy and tauopathy [14, 15]. Since tau-PET has not EMA approval in AD yet, lumbar puncture (LP) for both “A” and “T” biomarkers dosage is preferable, when possible, to the sole amyloid-PET, which should be limited to patients showing contraindications to LP [14, 15]. Plasma-based biomarkers will likely play a crucial role in detecting those individuals at higher risk for developing AD, allowing for earlier interventions [16]. They could even potentially be used for population-level screening for AD, granting access to cures also to those individuals living far from large urban centers.

The definition of the biological diagnosis parallels the profiling of the disease stage, as it is reasonable to assume that patients might obtain the greatest benefit from anti-Aβ immunotherapy if at an early stage of AD (i.e., prodromal or mild AD dementia), when amyloid is likely to exert more influence [30]. In addition, we think that there is no reason to exclude from immunotherapy patients diagnosed with non-amnestic phenotypes of AD.

A correct profiling of patients more suitable and in greater need for a prompt immunotherapy initiation requires a multidisciplinary approach and an extensive clinical–instrumental evaluation, which should include, besides “A” and “T” biomarkers definition, medical history collection, physical and neurological examination, complete neuropsychological evaluation, laboratory analysis, structural neuroimaging assessment (i.e., brain MRI), and brain FDG-PET. The integration of these results is indeed crucial for the definition of a personalized composite risk/benefit ratio, necessary to decide whether to propose or not an amyloid-lowering immunotherapy. First, prescribers will have to take into account the well-established risk factors for amyloid-related imaging abnormalities (ARIA) (i.e., ApoE ε4 status, MRI signs of cerebral amyloid angiopathy, cerebrovascular disease, dosage, and pharmacodynamic properties of an anti-Aβ mAb) [4, 31], but also the possible comorbidities (i.e., medical conditions other than AD, such as non-AD neurological disorders, bleeding disorders and antithrombotic therapy, cardiovascular diseases, history of recent cancer, renal or liver dysfunction, etc.). Another important point concerns prodromal AD patients at higher risk for a rapid conversion to a full-blown AD dementia, and thus in urgent need for a therapy [16, 28, 29].

## Reorganization of centers for dementia and cognitive decline in Italy

Cognitive impairment diagnosis and management in Italy are currently provided by more than 500 specialized memory clinics, also known as Centers for Dementia and Cognitive Decline (CDCDs) [32], specialized centers that provide multidisciplinary assessment, diagnosis, and treatment of patients with memory disorders, including dementia. According to a 2014 survey conducted by the Italian Ministry of Health [33], Italian CDCDs were characterized by heterogeneous resources in terms of diagnostic procedures (e.g., structural and functional neuroimaging, CSF AD biomarkers, etc.) and healthcare professionals involved (i.e., medical specialists, such as neurologists or geriatricians, administrative staff, neuropsychologists, rehabilitation specialists, etc.), with up to the 20% of centers declaring the inability to provide even a comprehensive neuropsychological assessment. Moreover, availability and accessibility of memory clinics varied significantly depending on the geography, resulting generally lower in southern regions and in rural and remote areas of our country. More than half of CDCDs were hospital-based (51%), 42% were territorial, and 7% were university centers, involving more than 1500 healthcare professionals in total, whereas waiting times for the first visit were generally lower than three months. Finally, the median number of patients attending each CDCD was 450. Since the approval of the Italian National Dementia Plan in 2014, great effort has been made by the Italian government to promote evidence-based healthcare interventions, to create a network of integrated services and to improve the access to these services for people with dementia [34]. With this as a starting point, the panel of experts deems mandatory the redefinition of dementia care setting in Italy and proposes here a possible solution, based on some considerations:

- To guarantee universal access to future therapies, competences and duties should be distributed to CDCDs based on a multilevel and increasingly complex organizational structure;
- Centers and community health system should be put into a network to guarantee the continuous multidimensional management and monitoring of the patient from the diagnosis and throughout the course of the treatment. The network will involve general practitioners (GPs), neurologists, geriatricians, psychiatrists, neuropsychologists/psychologists, neuroradiologists, laboratory doctors, nuclear medicine doctors, nurses, physiatrists, physiotherapists, speech language therapists, occupational therapists, other rehabilitation professionals and social workers;
- The need to define a biological diagnosis of AD has clear consequences on the organization of the diagnostic process. Indeed, since LP for the analysis of CSF biomarkers (or the execution of an amyloid-PET, when LP is contraindicated) is an essential requirement, it will be necessary to include the analysis of CSF biomarkers in the new national tariff as Essential Level of Assistance (LEA) (currently reimbursable only in the Region of Umbria);
- The high risk/benefit ratio of anti-Aβ immunotherapies requires a highly specialized diagnostic and a thorough exclusion criteria assessment, which should be dispensed by a specialist in neurology afferent to an expert memory clinic, ideally as the conclusion of interdisciplinary meetings for more complex cases;
- ARIA management will require standardized clinical and neuroradiological protocols in both routine and emergency clinical settings, together with the identification of appropriate neuroradiological centers to comply with the necessary high number of MRI scans and with the demanded highly specialized competences;
- Awareness raising campaign and specific continuous training for involved professionals and in accordance with their competences are further central aspects in tackle what will most likely be the health challenge of the twenty-first century.

In summary, the panel agrees on an organization of the CDCDs for the diagnosis of patients with AD into 3 levels of increasing complexity: community center, first-level center, second-level center. For each level, tasks and requirements have been defined as follows and as reported in Tables 1, 2, 3. Tables 1, 2, 3 also indicate, for each requirement, the parameters for verifying the appropriateness of the center.

**Table 1:** Community center

	Clinical evaluation	Global screening of cognitive performances	Neuropsychological evaluation	Structural neuroradiological examination	Biological diagnosis	Other examinations	Genetic analysis (ApoE status)
	Necessary	Necessary	Necessary	Necessary	Not necessary	Not necessary	Not necessary
TASKS What is the center to do?	Evaluation (medical history, identification of cardiovascular and neurodegenerative disease risk factors, visit). Exclusion of main differential diagnoses (particularly internal/iatrogenic secondary causes) Evaluation of co-pathologies Identification and activation of a local service network	Administration/interpretation of screening tests for cognitive impairment (MMSE, MOCA, mini-Cog, etc.) Application of functional scales (ADL, IADL, possible CDR) and anxiety/depression scales	Clinical-psychological interview. Administration/interpretation of tests for the evaluation of memory, attention, language, executive function, visuospatial functions	Request of brain CT in selected cases. Preferential request of brain MRI (with appropriate sequences). Results interpretation by exclusion of main differential diagnoses and evaluation of specific patterns (atrophy, cerebrovascular disease)			
REQUIREMENTS To adequately perform the indicated tasks	At least 1 neurologist/geriatrician/psychiatrist experienced in dementia	At least 1 neurologist/geriatrician/psychiatrist experienced in dementia	At least 1 neuropsychologist experienced in dementia	Access to a neuroradiology service with an MRI scanner (at least 1.5 T). Availability of brain MRI slots with appropriate sequences and in suitable times. At least 1 neuroradiologist experienced in dementia			
READINESS Parameters to verify appropriateness	Nr. neurologists/geriatricians/psychiatrists, hours/days per week per practitioner Nr. max patients in charge Network for local services	Nr. neurologists/geriatricians/psychiatrists, hours/days per week per practitioner Nr. max patients in charge	Nr. neuropsychologists, hours/days per week per practitioner Nr. max patients in charge	Nr. dedicated CT and/or MRI slots/month Nr. neuroradiologists with specific training, hours/days per week per practitioner Waiting list times			

*ADL* activities of daily living, *ApoE* apolipoprotein E, *CDR* clinical dementia rating scale, *CT* brain computer tomography, *iADL* instrumental activities of daily living, *MMSE* mini-mental State examination, *MOCA* Montreal Cognitive Assessment, *MRI* brain magnetic resonance imaging

**Table 2:** First-level center

	Clinical evaluation	Global screening of cognitive performances	Neuropsychological evaluation	Structural neuroradiological examination	Biological diagnosis	Other examinations	Genetic analysis (ApoE status)
	Necessary	Necessary	Necessary	Necessary	Necessary	Necessary	Possible
TASKS What is the center to do?	Evaluation (medical history, identification of cardiovascular and neurodegenerative disease risk factors, visit). Exclusion of main differential diagnoses (particularly internal/iatrogenic secondary causes) Evaluation of co-pathologies Identification and activation of a local service network	Administration/interpretation of screening tests for cognitive impairment (MMSE, MOCA, mini-Cog, etc.) Application of functional scales (ADL, IADL, possible CDR) and anxiety/depression scales	Clinical-psychological interview. Administration/interpretation of tests for the evaluation of memory, attention, language, executive function, visuospatial functions	Request of brain CT in selected cases. Preferential request of brain MRI (with appropriate sequences). Results interpretation and exclusion of main differential diagnoses and evaluation of specific patterns (atrophy, cerebrovascular disease)	LP execution according to standardized procedures and sending CSF to an external laboratory. PET request with amyloid tracer to be performed at the reference center Results interpretation	Test request (FDG-PET, DAT-SPECT, myocardial scintigraphy with I-123 MIBG) at referral center Results interpretation	Blood sampling
REQUIREMENTS to adequately perform the indicated tasks	Dedicated outpatient clinic At least 1 neurologist/geriatrician/psychiatrist experienced in dementia	Dedicated outpatient clinic At least 1 neurologist/geriatrician/psychiatrist experienced in dementia	Dedicated outpatient clinic At least 1 neuropsychologist experienced in dementia	Access to a neuroradiology service with an MRI scanner (at least 1.5 T). Availability of brain MRI slots with appropriate sequences and in suitable times. At least 1 neuroradiologist experienced in dementia	Appropriate facilities for LP. At least 1 neurologist experienced in dementia (for LP execution and results interpretation) Possibility to store the CSF sample until sent to the reference center Contact with a CSF analysis laboratory with quality standards and adherence to international guidelines	Contact with a nuclear medicine service with quality standards and adherence to international guidelines	Contact with genetics laboratory
READINESS Parameters to verify appropriateness	Nr. neurologists/geriatricians/psychiatrists, hours/days per week per practitioner Nr. max patients in charge	Nr. neurologists/geriatricians/psychiatrists, hours/days per week per practitioner Nr. max patients in charge	Nr. neuropsychologists, hours/days per week per practitioner Nr. max patients in charge	Nr. dedicated CT and/or MRI slots/month Nr. neuroradiologists with specific training, hours/days per week per practitioner Waiting list times	Nr. LP beds (available for 4–6 h) Nr. slots/month for CSF exams and amyloid-PET at referral centers Waiting list times	Nr. exams/month at referral centers Waiting list times	Nr. exams/month Waiting list times

*ADL* activities of daily living, *ApoE* apolipoprotein E, *CDR* clinical dementia rating scale, *CSF* cerebrospinal fluid, *CT* brain computer tomography, *DAT-SPECT* dopamine transporter single-photon emission computed tomography, *FDG-PET* F-18 fluorodeoxyglucose positron emission tomography, *iADL* instrumental activities of daily living, *LP* lumbar puncture, *MMSE* mini-mental State examination, *MOCA* Montreal Cognitive Assessment, *MRI* brain magnetic resonance imaging

**Table 3:** Second-level center

	Clinical evaluation	Global screening of cognitive performances	Neuropsychological evaluation	Structural neuroradiological examination	Biological diagnosis	Other examinations	Genetic analysis (ApoE status)
	Necessary	Necessary	Necessary	Necessary	Necessary	Necessary	Necessary (on-site)
TASKS What is the center to do?	Evaluation (medical history, identification of cardiovascular and neurodegenerative disease risk factors, visit) Exclusion of differential diagnoses, including causes of rapidly progressing cognitive impairment, rare causes of cognitive impairment, causes of cognitive impairment in juvenile cases Evaluation of co-pathologies	Administration/interpretation of screening tests for cognitive impairment (MMSE, MOCA, mini-Cog, etc.) Application of functional scales (ADL, IADL, possible CDR) and anxiety/depression scales	Clinical-psychological interview. Administration/interpretation of tests for the evaluation of memory, attention, language, executive function, visuospatial functions Administration of innovative cognitive tests (for investigated functions, for example social cognition) or for administration methods (telemedicine)	Request of brain CT in selected cases. Preferential request of brain MRI (with appropriate sequences). Result interpretation and exclusion of main differential diagnoses and evaluation of specific patterns (atrophy, cerebrovascular disease) through the application of qualitative or semi-quantitative methods of scoring	LP and CSF collection according to standardized procedures and PET execution with amyloid tracer On-site analysis Results interpretation Determination of new CSF biomarkers (NfL?)	Test execution (FDG-PET, DAT-SPECT, myocardial scintigraphy with I-123 MIBG) On-site analysis Results interpretation	Blood sampling
REQUIREMENTS to adequately perform the indicated tasks	Dedicated outpatient clinic At least 1 neurologist experienced in dementias in network with a multidisciplinary team	Dedicated outpatient clinic At least 1 neurologist experienced in dementia	Dedicated outpatient clinic At least 1 neuropsychologist experienced in dementia Tele-neuropsychology	Access to a neuroradiology service with an MRI scanner (at least 1.5 T). Availability of brain MRI slots with appropriate sequences and in suitable times. At least 1 neuroradiologist experienced in dementia	Appropriate facilities for LP. At least 1 neurologist experienced in dementia (for LP execution and results interpretation) Access to a laboratory medicine service with quality standards and adherence to international guidelines Availability of dedicated PET slots	Access to a nuclear medicine service with quality standards and adherence to international guidelines Availability of slots for FDG-PET, DAT-SPECT, I-123 myocardial scintigraphy	Availability of a genetics laboratory Neuro-genetic counseling clinic
READINESS Parameters to verify appropriateness	Nr. neurologists, hours/days per week per practitioner Nr. max patients in charge Nr. visits with multidisciplinary equipe	Nr. neurologists, hours/days per week per practitioner Nr. max patients in charge	Nr. neuropsychologists, hours/days per week per practitioner Nr. max patients in charge Nr. tele-neuropsychology visits Online reporting	Nr. dedicated CT and/or MRI slots/month Nr. neuroradiologists with specific training, hours/days per week per practitioner Waiting list times Online reporting	On-site certified laboratory for CSF biomarker analysis and nuclear medicine service with specific expertise Nr. LP beds (available for 4–6 h) Nr. slots/month for CSF exams and amyloid-PET Waiting list times Online reporting	Nr. exams/month at referral centers Online reporting	Nr. exam/month Nr. counseling visits/month

*ADL* activities of daily living, *ApoE* apolipoprotein E, *CDR* clinical dementia rating scale, *CSF* cerebrospinal fluid, *CT* brain computer tomography, *DAT-SPECT* dopamine transporter single-photon emission computed tomography, *FDG-PET* F-18 fluorodeoxyglucose positron emission tomography, *iADL* instrumental activities of daily living, *LP* lumbar puncture, *MMSE* mini-mental State examination, *MOCA* Montreal Cognitive Assessment, *MRI* brain magnetic resonance imaging

**Community center** (Table 1):

- Tasks
  1. Clinical evaluation, global screening of cognitive performances, clinical–psychological interview and neuropsychological evaluation (investigated functions: memory, attention, language, executive, and visuospatial functions).
  2. Request and interpretation of structural neuroimaging exams.
  3. According to patients characteristics, referral either to local services network or to a higher level center for biological diagnosis and possible amyloid-lowering therapies prescription.
- Requirements
  1. At least 1 neurologist/geriatrician/psychiatrist + 1 neuropsychologist with expertise in dementia evaluation.
  2. Access to a neuroradiology equipped with a (at least) 1.5 T MRI scanner and a neuroradiologist with expertise in evaluation of dementia cases.
  3. Close contact with local services and with the reference centers for biological diagnosis and therapy prescription.

**First-level center** (Table 2):

- Tasks
  1. Clinical evaluation, global screening of cognitive performances, clinical–psychological interview, and second-level neuropsychological evaluation (investigated functions: memory, attention, language, executive and visuospatial functions, and social cognition).
  2. Request and interpretation of structural neuroimaging exams.
  3. Biological diagnosis of AD, obtained by performing LP on site and by sending the CSF to a reference center/external laboratory for biomarkers analysis, or alternatively by requesting an amyloid-PET to a reference center.
  4. Request and interpretation of other instrumental tests performed at the reference center (for example, EEG, FDG-PET, DAT-SPECT, and myocardial scintigraphy).
  5. Activation of local services network.
  6. (Possibly) Blood sampling for genetic analysis (ApoE status).
- Requirements
  1. Dedicated outpatient clinic.
  2. At least 1 neurologist/geriatrician/psychiatrist + 1 neuropsychologist with expertise in evaluation of dementia cases.
  3. At least 1 neurologist in case of LP.
  4. Availability on site of a (at least) 1.5 T MRI scanner and of a neuroradiologist with expertise in evaluation of dementia cases.
  5. Availability of facilities for LP.
  6. Contact with reference laboratory medicine service (for CSF analysis) and certified nuclear medicine service.
  7. (Possibly) Contact with certified genetics laboratory.

**Second-level center** (Table 3):

- Tasks
  1. Clinical evaluation for the identification also of rare causes of cognitive decline, rapidly progressive dementias and early-onset variants; global screening of cognitive performances, clinical–psychological interview and neuropsychological assessment including innovative (e.g., social cognition) and multimodal (e.g., telemedicine, virtual reality) testing.
  2. On-site execution and interpretation of all diagnostic procedures
    Structural neuroimaging,
    LP and CSF analysis, amyloid-PET,
    Other tests (e.g., EEG, FDG-PET, DAT-SPECT, myocardial scintigraphy),
    ApoE status definition.
- Requirements
  1. Dedicated outpatient clinic.
  2. At least 1 neurologist and 1 neuropsychologist with expertise in evaluation of dementia cases.
  3. Multidisciplinary team and network of diagnostic services.
  4. Availability on site of a (at least) 1.5 T MRI scanner and of a neuroradiologist with expertise in evaluation of dementia cases.
  5. Availability of facilities for LP.
  6. Availability on site of a certified laboratory medicine service (for CSF analysis) and of a certified nuclear medicine service (defined slots).
  7. Availability on site of a certified laboratory for genetic assessment (ApoE status).
  8. Telemedicine.

Moreover, the panel agrees on the tasks and requirements necessary for a center to apply for the prescription of anti-Aβ mAbs (Table 4). The prescribing center, in addition to carrying out the activities envisaged for a second-level center, must be equipped for the administration of new therapies, patients follow-up and the management of any side effects, including emergent ARIAs.

**Table 4:** Prescriber center

	Therapeutic indications evaluation	Pharmacy service	Medication administration	Management of medium- and long-term adverse effects	Clinical follow-up	MRI follow-up
	Necessary	Necessary	Necessary	Necessary	Necessary	Necessary
TASKS What is the center to do?	Evaluation of inclusion criteria (disease stage, comorbidity, MRI)	Drug preparation and dispensing (as per specific indication)	Drug administration (in particular ev, and following the indicated timing) Short-term adverse event management	Emergency specialist evaluation on-site or remotely (visit, review of examinations, treatment of side effects)	Clinical follow-up (neurological and cognitive) according to drug indications	MRI scans planning according to drug indications Evaluation of images and report
REQUIREMENTS To adequately perform the indicated tasks	Dedicated outpatient clinic At least 1 neurologist, 1 neuropsychologist, 1 case manager with specific training	Internal pharmacy service At least 1 experienced pharmacist	Dedicated outpatient clinic with chairs/bed for infusion and for subsequent monitoring (as per specific indications) At least 1 neurologist and 1 nurse with specific training	Emergency department access for acute and severe symptoms Daytime telephone availability (at least 8 h, 5/7 days per week) of qualified personnel Neurological re-evaluation and/or teleconsultation programmable in a short time (priority line) Possibility of a dedicated cell phone line for daytime hours 7/7 days Includes neuroradiological follow-up requirements (see specific column)	Dedicated outpatient clinic At least 1 neurologist, 1 neuropsychologist, 1 case manager with specific training	Access to a neuroradiology service with MRI scanner (at least 1.5 T). At least 1 neuroradiologist with specific training for ARIA Possibility of rapid consultation with a neuroradiologist (also teleconsultation) Multidisciplinary neurologist/neuroradiologist meetings
READINESS Parameters to verify appropriateness	Nr. neurologists, Nr. neuropsychologists, Nr. case managers. Hours/days per week per practitioner Nr. max patients in charge in the dedicated outpatient clinic Access to training courses for various professionals	Dispensed drug/month	Nr. max patients in charge in the dedicated outpatient clinic Nr. chairs/bed for infusion (availability in horus) per month. Nr. neurologists, Nr. nurses with specific training Access to training courses for various professionals	Possibility of urgent access to the emergency department Existence of an accessible telephone line and nr. of qualified personnel (nurses or case managers) to answer (at least 8 h, 5/7 days) Access to training courses for various professionals Includes readiness for neuroradiological follow-up (see specific column)	Nr. neurologists, Nr. neuropsychologists, Nr. case managers. Hours/days per week per practitioner Nr. max patients in charge in the dedicated outpatient clinic (based on the frequency of visits foreseen as per the indications of the drug) Access to training courses for various professionals	Nr. dedicated MRI slots for regular follow-up (according to drug indication) Nr. neuroradiologists with specific training Possibility of performing extra MRI scans in a timely manner

*ARIA* amyloid-related imaging abnormalities, *MRI* brain magnetic resonance imaging

## Prescriber center

- Tasks
  1. Evaluation of specific drugs indications and contraindications
  2. Preparation and dispensation of medication through a pharmacy service
  3. Drug administration and monitoring/treatment of short-term adverse events
  4. Management of follow-up, ensuring possible short-term clinical evaluation, blood chemistry screening and MRI scan
  5. Monitoring and treatment of adverse events in the medium and long term (in person or remotely)
- Requirements
  1. Availability of a pharmacy service
  2. Availability of outpatient clinic dedicated to medication intravenous infusion
  3. The presence of:
    At least 1 neurologist to define eligibility and actively monitor the drug administration
    At least 1 nurse with specific training
    At least 1 case manager for organizing the patient’s therapeutic pathway and collecting direct requests to the center
  4. Ensure daytime telephone availability through a dedicated telephone line (at least 8 h for 5 days) and a 24 h contact for emergencies
  5. Emergency room
  6. Neuroradiology service with a (at least) 1.5 T MRI scanner and neuroradiologists expert in the evaluation of dementia and ARIA cases and guaranteeing the number of MRI exams necessary per patient, even in urgent conditions
  7. Possibility of teleconsultation/telemedicine
  8. Training and updating programs dedicated to the center professionals

The panel also considers mandatory the identification of readiness parameters for assessing the application of each CDCD within the proposed framework of diagnostic/prescriber centers (Tables 1, 2, 3, 4). Before this, their evaluation will also be crucial for policy makers and regulatory agencies to ascertain the very realistic lack of human and structural resources in our country, as in the most western countries, in facing the authorization of disease-modifying therapies for AD. [35]

## Conclusion

Beyond controversies regarding the actual efficacy of amyloid-lowering therapies, based on significantly positive biological and clinical results of trials testing lecanemab and donanemab, which granted the recent accelerated approval of Leqembi™, it is reasonable to expect their marketing authorization in Europe in the next two/three years (as soon as 2024 for early market access of lecanemab in Italy, since the application to EMA was made in January, 2023).

Notwithstanding the contraindications, the number of potentially treatable patients in Italy will be tens of thousands, as suggested in a recent Italian study and in a similar French paper [2, 27]. To successfully face such a breakthrough change in AD treatment, a quick reorganization of Italian CDCDs will be necessarily required, together with the definition of clinical and neuroradiological management protocols. We suggest here a possible solution, including 3 levels of increasing complexity centers for the diagnosis and the continuous care of AD patients, and proposing the requirements for a center to apply for the prescription of disease-modifying therapies. Level-specific parameters are also suggested for ascertaining the readiness of each center and of the Italian Health system in general, to put light on the resources which have to be allocated to face the enormous change required by the approval of anti-Aβ mAbs. Moreover, we think that upcoming drugs should be dispended by a neurologist after the ascertainment of the abnormality of both amyloidopathy and tauopathy biomarkers and based on the patient-specific risk/benefit ratio. In the next future, as the research continues, plasma-based biomarkers may be inserted in the diagnostic work-up of AD and their dosage used to save on costs and resources for more invasive and expensive procedures, such as LP or amyloid-PET, in patients showing normal plasmatic levels. Ideally, the request and the interpretation of plasma-based biomarkers should be executed already at community centers, but at least until their use becomes routine in centers deputed to biological diagnosis of AD, it will be realistically limited to first-level and second-level centers. Finally, the approval of disease-modifying therapies will affect AD care networks, requiring a close and continuous contact between specialists and general practitioners, to provide wider access for the Italian population to an early-stage biological diagnosis of AD and to guarantee the best monitoring to treated patients.
